# Virulence profile of pathogenic yeasts from snakes: Alternative ways for antifungal strategies

**DOI:** 10.1371/journal.pone.0318703

**Published:** 2025-03-12

**Authors:** Iniobong Chukwuebuka I. Ugochukwu, Jairo Alfonso Mendoza-Roldan, Mara Miglianti, Natalizia Palazzo, Amienwanlen Eugene Odigie, Domenico Otranto, Claudia Cafarchia

**Affiliations:** 1 Department of Veterinary Medicine, University of Bari Aldo Moro, Valenzano, Bari, Italy; 2 Department of Veterinary Pathology, University of Nigeria, Nsukka, Nsukka, Nigeria; 3 Department of Veterinary Clinical Sciences, City University of Hong Kong, Hong Kong, SAR China; Cleveland Clinic Lerner Research Institute, UNITED STATES OF AMERICA

## Abstract

Reptiles may act as reservoirs or spreaders of potential pathogenic microorganisms including *Candida* yeasts. While the epidemiology of yeast species has been thoroughly studied, the virulence profile of isolated species is not well investigated. Therefore, this study aimed to assess the haemolytic, phospholipase, lipase activities and biofilm formation of yeasts isolated from the cloacal swabs of venomous snakes from Marrakech, Morocco (Group I, n = 40) and from non-venomous snakes from Cocullo, Italy (Group II, n = 32). All the isolated yeasts from Group 1 showed low production of lipase (Lz ≥  0.90) and haemolysin (Hz ≥ 0.90), and only 35% of them were low phospholipase (Pz) producers (Pz > 0.90). In contrast, all the yeasts from Group 2 produced enzymes and more than 62% produced high amounts of enzymes (Pz ≤  0.64; Lz ≤  0.69; Hz ≤ 0.69). Data show that yeasts from snakes were able to produce virulence factors, which vary according to the yeast species and the hosts or their origin, thus suggesting the potential role of snakes in harboring and spreading pathogenic yeasts in the environment. Since the virulence profile was lower in venomous snakes than that in non-venomous ones, we discussed that it may be affected by the venom composition. This will pave the way for fungal infection control, alternative to antifungal drugs in order to overcome resistance phenomena.

## Introduction

Fungal infections poses an emerging global health threat, prompting the World Health Organization (WHO) to release the first list of health-threatening fungi to pilot research and public health interventions [1]. Importantly, wildlife is a major source of infectious diseases, including mycoses [2,3], many of them of zoonotic concern worldwide [3,4]. In the last decades, reptiles have attracted the interest of the scientific community, for their role as spreaders of bacteria (*Salmonella* spp., *Vibrio* spp.), viruses (*arboviruses*) and fungi (*Candida* spp.) [5–8]. In addition, many wild animal species (e.g., Cheetahs, Koalas, Lizards, Deer, Dolphins, Elk, Porpoises, Sea Lion, Baleen whales, Wild Boars, Snakes) were considered as sentinels for human/animal pathogenic microorganisms. For example, in our previous study focusing on snakes of different origin we have isolated pathogenetic yeasts including fungal species listed among WHO Critical and High Priority group of fungal pathogens [9]. Since the yeast species community as well as their antifungal profile varied according to animal origin and lifestyle reflecting the epidemiology of human yeast infections in the same geographical areas, we have suggested that these animals including snakes should be considered as sentinels for human/animal pathogenic microorganisms and bio-indicators of environmental quality [6–8,10,11]. As a matter of fact, yeasts belonging to the genera *Candida, Cryptococcus, Geotrichum, Rhodotorula, Debaryomyces, Meyerozyma* and *Trichosporon* were frequently isolated from wild animals [6,8,11–13]. All of the above yeasts are considered a public health concern of emerging importance, due to the increased number of their infection in humans and animals [14,15]. In particular, yeasts of both endogenous (e.g., *Candida albicans,* *Pichia kudriavzevii, Candida parapsilosis*) or exogenous origin (e.g., M*eyerozyma guilliermondii, Candida fermentati, Candida lusitaniae, Cryptococcus spp., Trichosporon spp., Rhodotorula spp*.) may induce cutaneous and systemic diseases in humans and animals [16–22]. In addition, their low antifungal drug susceptibility has been considered, the major cause of outbreaks [23]. These yeasts can persist within the host without causing diseases, spread in the environment through the feces, eventually acquiring virulence determinants, such as antifungal resistance [24–26]. Indeed, there is a growing concern that these yeast species might acquire virulence determinants and antimicrobial resistance when they move from one niche or location to another [27,28].

Virulence factors are key determinants of pathogenicity in yeast, enabling them to cause infections in humans and animals [24–26,29–34]. In particular, one group of virulence factors causes colonization to take place, or the initiation of an infection, whilst the other group helps to spread the infection [34]. The most studied virulence traits of yeast are adhesion to host tissues and surface of medical devices, biofilm formation, extracellular hydrolases production, phenotypic switching and thigmotropism and for *Cryptococcus* spp., capsule formation and melanin production. Adhesion to the tissue is the primary and most important stage in the process of yeast colonization and infection helping yeast cells to penetrate, disseminate and persist in host tissues. Biofilms are surface associated microbial communities firmly fixed within an extracellular matrix, which limits the penetration of an antifungal agent and protects the yeast cells from host defense mechanism [32,33]. The ability to form biofilm as well as its structure is yeast species dependent thus contributing in the enhancement of the virulence of the specific yeast species [32,33]. Extracellular hydrolytic enzymes facilitate the invasion of host tissue by damaging host cell membrane thus playing an important role in the pathogenesis of yeast infections. Phospholipases, aspartyl proteinases, lipases and hemolysins are the secreted hydrolases most commonly implicated in the yeast pathogenicity [24–26,29,30]. Finally the production of hyphae or thigmotropism, described mainly in *C. albicans*, *C. dubliniensis* and *C. tropicalis* are considered mechanisms of virulence facilitating tissue invasion and resistance by phagocytosis. Polysaccharide capsule formation and melanin production, as seen in *Cryptococcus* species, protects the yeast against host immune responses [35]. These virulence factors working in combination allow yeast to inhabit host tissues, avoid immune responses, and withstand treatment, thus complicating both diagnosis and therapy [36]. However proteinases, lipases, phospholipases as well as biofilm formation were the most studied virulence factors, and they well evaluated from many yeast species that cause human and animal infections [24–26,29,30,34].

Although the epidemiology of *Candida* or non-*Candida* species has been thoroughly studied [37], the virulence profile of yeasts isolated from wild animals has not been fully elucidated. In particular in our previous study we observed that the fungal community as well the occurrence of pathogenic yeasts in the cloaca of snakes varied according to venomousity of the animals suggesting a role of the venom in selecting pathogenic yeast populations. Therefore, this study aimed to assess the production of i) hydrolytic enzymes such as phospholipase, lipase and hemolysin and ii) the biofilm formation of different yeast species isolated from the cloacal swabs of venomous and non-venomous snake species from Marrakech, Morocco and Cocullo, Italy.

## Materials and methods

### Yeast strains

**Fig 1:**
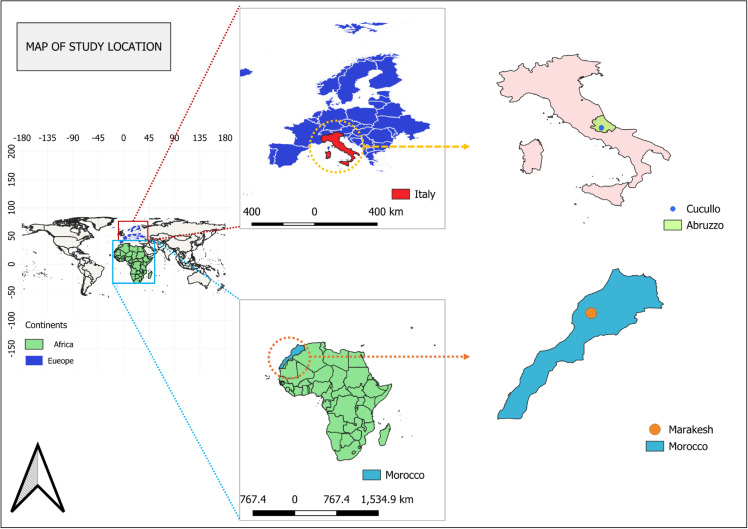
Map showing the sites where snake cloacal samples were obtained. Map prepared using QGIS software—Buenos Aires version (link of the XYZ tile: https://tile.openstreetmap.org).

A total of 72 yeast strains previously isolated and molecularly identified from the cloacal swabs of venomous snakes from Marrakech, Morocco as indicated in Fig 1 (Group I, n = 40) and from non-venomous snakes from Cocullo, Italy as indicated in Fig 1 (Group II, n = 32) were employed in the study (Table 1). Animals were apparently healthy and collected under the frame of previous studies assessing the human-reptile-pathogens interface [8,38,39]. Briefly, Group 1 is composed by species of snakes that properly represent the typical Moroccan herpetofauna (22 species of snakes, being eight considered dangerous), associated to the traditional practices and snake charming. Given that snakes are pivotal for this ancestral custom, the species involved are represented by the more eye-catching ones such as the Egyptian cobras, given their unique display of warning behavior. In addition, species of highly venomous snakes such as puff adders, as well as mildly venomous ones, as in the case of Montpellier snakes, are also frequently used, with even the latter species handed to tourists. In Morocco, snakebites are of important concern mainly in children from rural areas of the northern-central regions, with an average of 218 cases per year, being chiefly caused by vipers. Conversely, snakebite envenomation in charmers is underreported, most likely due to the possibility of risking their livelihood if they report snakebites [38]. On the other hand, Group 2 is characterized by non-venomous species with the four-lined snake being the most captured given its essential role within the “*festa dei serpari”*, once these large snakes are placed on top of the statue of *San Domenico.* Overall, in Italy five species of venomous vipers are present, with *Vipera aspis* and *Vipera ursini* also occurring in the Abruzzo region, where the snake ritual takes place. However, snakebites are underreported and sporadic, with three deaths reported in a four-year period (1980–1984). Indeed, given their shy and fossorial nature, as well as their distinctive morphological features, vipers have been never captured for the snake ritual and there are no reports of snake catchers from the region being bitten by venomous snakes [39].

**Table 1:** Snake species sampled in Marrakech, Morocco (Group I) and Cocullo, Abruzzo, Italy (Group II) and relative isolated yeast species.

Sampling Location	Species	Yeast species (Number of isolates)
Group I (Marrakech, Morocco)	*Naja haje legonis* ***	*Candida tropicalis* (4)*, Diutina catelunata* (3)
	*Bitis arietans* ***	*Candida parapsilosis*; *Candida tropicalis* (7)*; Exophiala dermatitidis* (2)*; Meyerozyma caribbica* (2)*; Trichosporon asahii* (10)*; Wickerhamiella pararugosa* (1).
	*Malpolon monspessulanus* **	*Rhodotorula mucilaginosa* (2)*; Clavispora lusitaniae* (1)*; Trichosporon asahii* (6)
Group II (Cocullo, Abruzzo, Italy)	*Elaphe quatuorlineata* *	*Cryptococcus neoformans* (4)*; Debaryomyces hansenii* (4)*; Rhodotorula mucilaginosa* (4)
	*Zamenis lineatus* *	*Debaryomyces hansenii* (4)*; Metahyphopichia silvanorum* (4)*; Meyerozyma guilliermondii* (4)
	*Hierophis viridiflavus* *	*Metahyphopichia silvanorum* (4)

*Non-venomous;

**Mildly venomous and

***Highly venomous.

The strains were stored at −  80 °C at the Department of Veterinary Medicine, University of Bari (Italy). To ensure purity and viability, each strain was sub-cultured at least twice onto Sabouraud dextrose Agar (SDA, Liofilchem, Italy) incubated at 35 °C for 24–48 h, prior testing the virulence profiles.

### Ethics statement

This research followed relevant international, national, and institutional guidelines regarding the handling of animals. In particular, the procedures for collecting samples from Morocco were approved by the Office National de Sècurité Sanitaire des Produits Alimentaires in the Kingdom of Morocco, under the authorization number 23355ONSSA/DIL/DPIV/2022. Whereas, the protocols of snake sampling, handling, and capture by Serpari, in Italy, was allowed by the National authorizations (National law DPR 357/97) and approved by the Italian Ministry of Environment (n. 16271/2023 PNM and 79052/2023 PNM) as previously reported [8].

### Enzymatic activity assays

#### Preparation of the yeast suspensions.

A loop of the pure stock culture of each yeast strain was cultured onto SDA and incubated at 32°C for 72h (*Cryptococcus neoformans*) or 37°C for 48h (other yeasts). Pure colonies were transferred into sterile distilled water and adjusted to an optical density of 1.3 using a turbidimeter (DEN-1McFarland Densitometer, Biosan) that was equivalent to 1.5 ×  10^7^ colony forming units (CFU)/ml as validated by quantitative plate count CFU in SDA using standard procedures [28,40].

#### Haemolytic activity.

**Fig 2:**
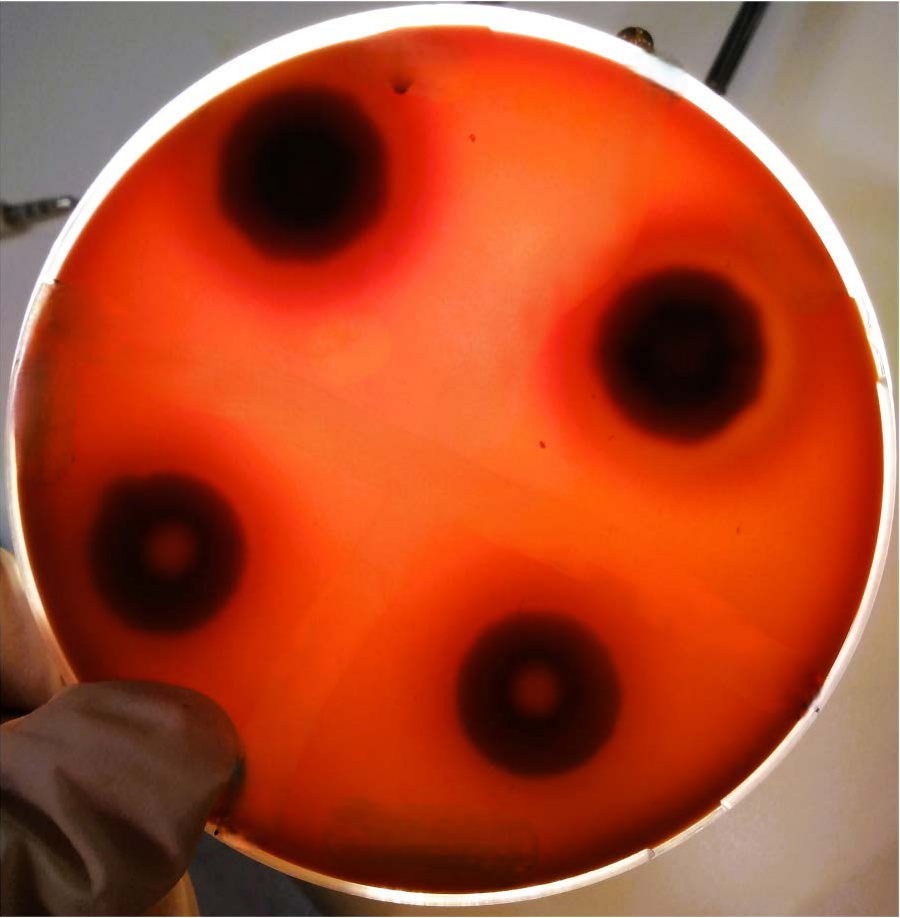
Haemolytic activity of *Candida tropicalis* strains (CD1849 and CD 1850).

The haemolytic activity of yeast species were assessed by the blood plate assay using SDA supplemented with 7% sheep blood and 3% glucose and adjusted to a pH of 5.6 [41]. A total of 10 μl yeast suspension was inoculated in duplicates onto the media and incubated at 37°C for 5 days. After incubation, a distinct translucent halo around the inoculation site indicated positive haemolytic activity [42]. The ratio of the diameter of the colony (a) by that of the translucent zone and that of the diameter of the colony (b) was used as value of the haemolytic index (Hz value). Each strain was tested in duplicates (Fig 2), and the experiments were repeated three times in different days. The Hz values were reported as average of the values registered. According to the Hz index, the following activity ranges were established: high, Hz ≤  0.69; moderate, Hz =  0.70– 0.89; weak, Hz =  0.90–0.99; none, Hz =  1 [43].

#### Phospholipase activity.

**Fig 3:**
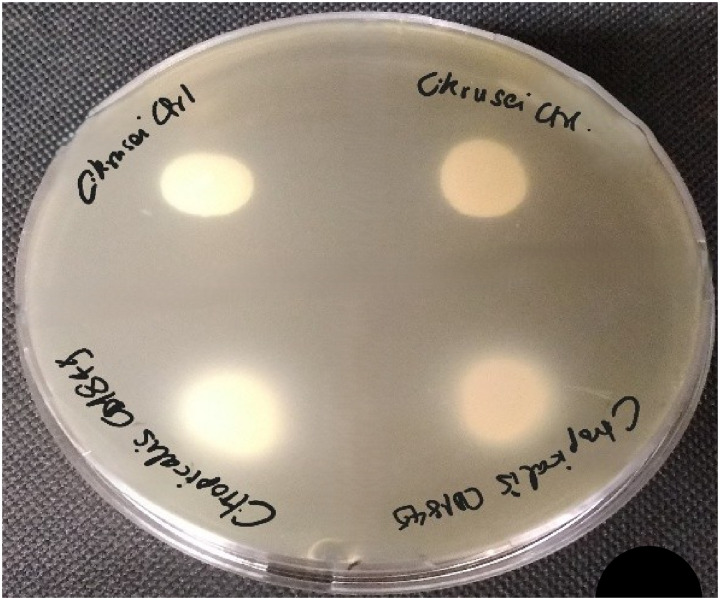
Phospholipase activity of *Candida tropicalis* (CD1849) and *Candida krusei.*

The production of phospholipase was assessed using the egg-yolk plate method as previously described [24,44]. A total of 10 μl yeast suspension was inoculated in duplicates onto egg-yolk agar media plates and incubated at 32°C for 5 days. The formation of a precipitation zone (i.e., opaque halo) around the yeast colony was considered as an indicator for enzyme production. Phospholipase activity was expressed as Pz, which represents the ratio between colony diameter and total diameter plus zone of precipitation [45]. Each strain was tested in duplicates (Fig 3), and the experiments were repeated three times in different days. The results were expressed as the average of the values obtained. According to the Pz index, the following activity ranges have been established: strong enzymatic production: Pz ≤ 0.64, moderate: Pz =  0.64–0.89; weak: Pz =  0.90–0.99; none, Pz =  1

#### Lipase activity.

**Fig 4:**
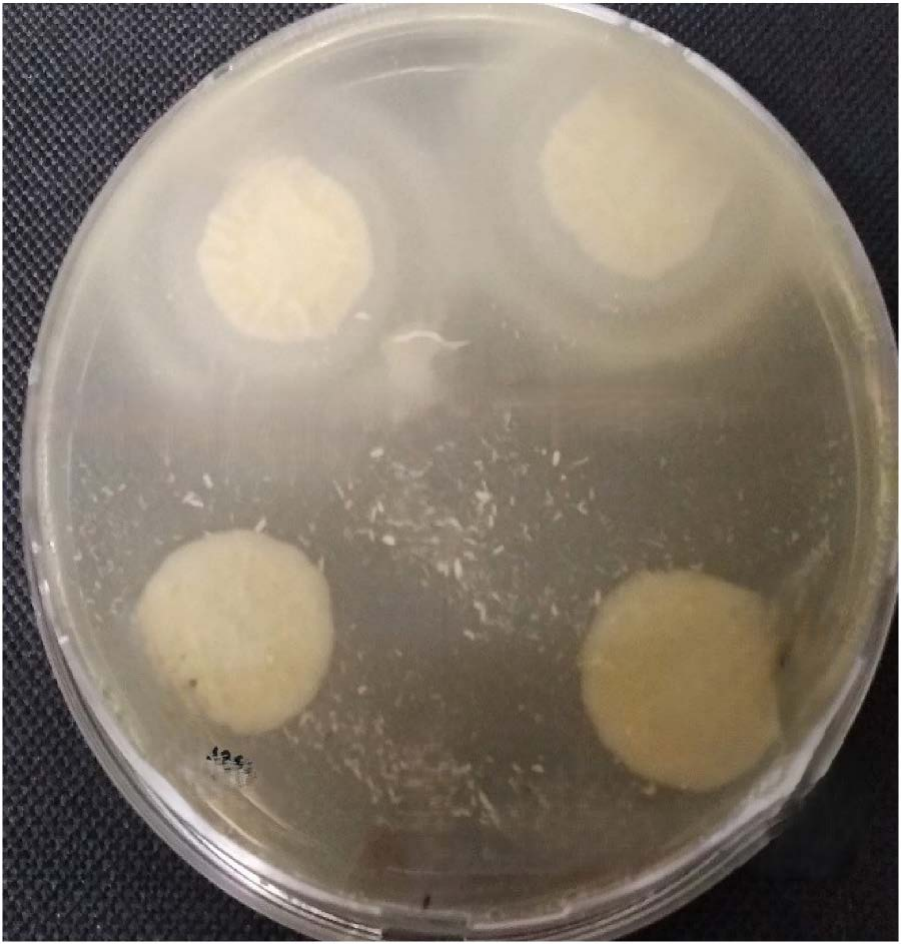
Lipase activity of *Candida tropicalis* strains (CD1849 and CD 1850).

The lipase activity (Lz) was assessed, following standard procedures as previously described [46]. Briefly, 10 µ L of each strain suspension was cultured in a sterile petri disk containing a lipid medium (i.e., peptone 1%, sodium chloride 5%, calcium chloride 0.01%, and agar 2%, plus 1% tween 80) and incubated at 32°C for 5 days. A zone of precipitation around the colony indicated lipase production. The production of lipase (Lz) is expressed as a ratio of diameter of a colony to the total diameter plus zone of precipitation. Each strain was tested in duplicates (Fig 4), and the experiments were repeated three time in different days. The results were expressed as the average of the values obtained. The ranges of activity according to the Lz index were established as follows: high: Lz ≤  0.69; moderate, Lz =  0.70– 0.89; weak, Lz =  0.90–0.99; none, Lz =  1 [46].

### *In vitro* biofilm formation

The biofilm production was tested using a modified standard method [47–49]. Briefly, yeast cells were suspended in RPMI 1640 broth (Sigma, USA) and the suspension was adjusted to the concentration of 1 ×  10^6^ cells/mL. Subsequently, 100 μL of the inoculum were transferred to 96-well flat-bottomed polystyrene plates containing 100 μL of RPMI 1640 broth. The plates were incubated at 35°C for 24 hours for *Candida* spp. and other isolated yeast species and at 37°C for 72 for *C. neoformans*. Wells containing only RPMI 1640 broth without inoculum were used as negative control. After incubation, the supernatant was carefully aspirated, and non-adherent cells were removed by gently washing twice using sterile phosphate buffer saline solution (PBS). Subsequently, biofilms were measured using the 2,3-bis-(2-methoxy-4-nitro-5-sulfophenyl)-2H-tetrazolium5-carboxanilide (XTT) reduction assay. In particular, the washed biofilm cells were incubated for 3 hours at 37°C with a solution of 1 mg/ml of XTT and 1μM of menadione in PBS. The volume was then transferred into a new 96-well plate, which was read soon after using a Spectrophotometer at 490 nm. Each test was performed in duplicates and the experiments were repeated three time in different days. The results were expressed as the average of the obtained values (i.e., mean values of optical density-OD) [50].

### Statistical analysis

Statistical analysis was performed in Python (version 3.9.18). We utilized several core libraries and their respective dependencies in the Python programming environment, which included Pandas for high-performance data analysis, Numerical Python (NumPy) for array operations, Matplotlib and Seaborn for interactive and statistical data visualizations, as well as Scikit-learn and SciPy for data preprocessing, and evaluation [51,52]. The repeated measures ANOVA (rm-ANOVA) and pairwise post hoc comparisons where a statistical significance was performed using pingouin and statsmodels. A critical p-value threshold of p ≤  0.05 was applied to determine statistical significance. The rm- ANOVA was most appropriate as it accounted for the dependence between repeated measures at different time points within the dataset while the Tukey’s Honestly Significant Difference (HSD) test, a commonly used post-hoc test that adjusts for the family-wise error rate, was used to identify specific group comparisons where such significant difference was found.

## Results

**Fig 5:**
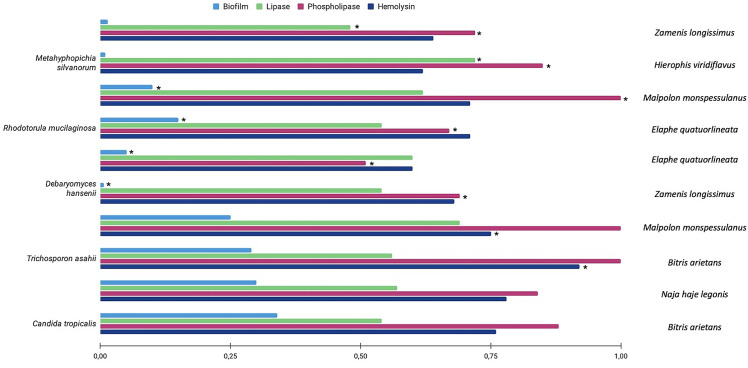
Comparison of virulence profiles of yeast spp. isolated from different snakes.

All the isolated yeasts from Group 1 produced haemolysin and lipase whereas only 35% produced phospholipase (Table 2; Figs 2,3,4). However, the amount of produced enzymes were classified as very low, varying according to the isolated yeast species (Table 2). In Group 1, *Exophiala dermatitidis* showed the highest haemolytic (Hz = 0.52), phospholipase (Pz = 0.5) and lipase (Lz = 0.5) activities. Whereas *Diutina catelunata* showed the high lipolytic (Lz = 0.46) and haemolytic activities (Hz = 0.53). Only some strains of *Candida tropicalis* and *E. dermatitidis* were phospholipase producers. Conversely, all yeasts from Group 2 produced enzymes, and many of them (e.g., 62%) in high quantity (Table 2). In particular, *C. neoformans* produced the highest haemolytic and lipase activities (Hz = 0.61; Lz = 0.53), and *Meyerozyma guilliermondii* the highest phospholipase (Pz = 0.60) activities. All the yeast species produced biofilm varying according to the isolated yeast species (Table 2). The yeast enzymatic activities recorded varied according to snake species (Fig 5), with the lipase and phospholipase activities of *Metahyphopichia silvanorum* being highest in strains isolated from *Zamenis longissimus* compared to those isolated from *Hierophis viridiflavus*. The biofilm formation and phospholipase activity of *Rhodotorula mucillaginosa* and *Debaryomyces hansenii* were greater in strains isolated from *Elaphe quatuorlineata* and *Z. longissimus*, respectively, than from *Malpolon monspessulanus*. In addition, the haemolysin activity of *Trichosporon asahii* was greater in strains isolated from *Malpolon monspessulanus* than that from *Bitis arietans*. No statistical differences in virulence factors of *C. tropicalis* were registered among the strains isolated from *B. arietans* and *Naja haje legionis*.

**Table 2:** Virulence factors of yeast species isolated from cloacal swabs of snakes from Marrakech, Morocco and Cocullo, Abruzzo, Italy.

Sampling Location	Yeast Species (Number of isolates)	Hemolysin Mean±SD	Phospholipase Mean±SD	Lipase Mean±SD	Biofilm Mean±SD
**Group I (Marrakech, Morocco)**	*Candida parapsilosis* **(n = 2)**	0.88 ± 0.16^a,b,^	1 ± 0^a,b^	0.55 ± 0.09^a^	0.65 ± 0
	*Candida tropicalis* **(n = 11)**	0.77 ± 0.15^c,d,e^	0.87 ± 0.13^a,c,d,e,f,g,l^	0.55 ± 0.07^b^	0.33 ± 0.04
	*Clavispora lusitaniae* **(n = 1)**	0.70 ± 0.02^f^	1 ± 0^l,m^	0.75 ± 0.22^a,b,c,d,e,f,g^	0.23 ± 0
	*Diutina catelunata* **(n = 3)**	0.53 ± 0.03^a,c,g,h,I,j^	1 ± 0^c^	0.46 ± 0.01^c^	0.11 ± 0
	*Exophiala dermatitidis* **(n = 2)**	0.52 ± 0.01^b,d,j,k,l,j^	0.5 ± 0^b,d,h,i,j,k,m^	0.5 ± 0.05^d,e^	0.22 ± 0
	*Meyerozyma caribbica* **(n = 2)**	0.79 ± 0.16^g,j^	0.9 ± 0.14^h^	0.53 ± 0.07^f^	0
	*Rhodotorula mucilaginosa* **(n = 2)**	0.71 ± 0.04^m^	1 ± 0^e,i^	0.62 ± 0.02	0.10 ± 0
	*Trichosporon asahii* **(n = 16)**	0.85 ± 0.13^h,k^	1 ± 0^f,j^	0.61 ± 0.14	0.27 ± 0.18
	*Wickerhamiella pararugosa* **(n = 1)**	0.96 ± 0.05^e,f,i,l,m,^	1 ± 0^g,k^	0.53 ± 0.02^g^	0.06 ± 0
	**Pos/Tot (%)**	40/40(100%)	14/40 (35%)	40/40 (100%)	38/40 (95%)
	**High production/ pos(%)**	5/40(12.5%)	2/40 (5%)	39/40 (97.5%)	–
**Group II (Cocullo, Abruzzo, Italy)**	*Cryptococcus neoformans* **(n = 4)**	0.61 ± 0.04^A^	0.75 ± 0.11^A^	0.53 ± 0.10	0.053 ± 0.016^A,B,C^
	*Debaryomyces* *hansenii* **(n = 12)**	0.66 ± 0.10	0.64 ± 0.13^A,C^	0.56 ± 0.06	0.02 ± 0.02^D,E^
	*Metahyphopichia silvanorum* **(n = 8)**	0.63 ± 0.03^B^	0.79 ± 0.07^C,D^	0.6 ± 0.18	0.0129 ± 0.007^B,F,G^
	*Meyerozyma guilliermondii* **(n = 4)**	0.61 ± 0.02^C^	0.60 ± 0.004^B,D^	0.52 ± 0.056	0.15 ± 0^A,D,F^
	*Rhodotorula mucilaginosa* **(n = 4)**	0.71 ± 0.06^A,B,C^	0.67 ± 0.07	0.54 ± 0.12	0.15 ± 0.038^C,E,G^
	**Positive/Total (%)**	32/32(100%)	32/32(100%)	32/32(100%)	32/32(100%)
	**High production/ Pos(%)**	28/32(87.5%)	20/32(62.5%)	32/32(100%)	–

The same superscript letters per column indicate statistically significant differences (p ≤  0.05).

## Discussion

The results of this study suggest that yeasts isolated from snakes are able to produce virulence factors, which varied according to their species, host and/or geographical origin, overall suggesting the potential role of snakes in harbouring and spreading pathogenic yeasts in the environment. It is well known that wild animals may act as reservoirs of yeasts which varied according animal life style and origin [6,8,11,14,25,53]. In addition, the cloaca yeast population is usually retrieved in the faeces of animals, thus suggesting that these animals might act both as reservoirs and spreaders of pathogenic yeasts in the environment through their faeces [6,11,12,14,28,54]. In our previously published study we have demonstrated that mycobiota composition of cloaca of different snake species varied according to animal species and origin and were positively correlated with the etiology and epidemiology of human and animal infection in the same geographical area [8]. The presence of yeasts species in the cloaca of these animals seems to be due to the fact that these microorganisms were acquired from the environment thus acting as a colonizer of the skin and/or of the gastrointestinal tract [8,53]. This evidence was well demonstrated by the isolation of *Candida auris* from cloaca swab of Egyptian cobra [7]. Since most species of yeasts isolated from snakes have already been established as causative agents of life-threatening infections characterized by high mortality in immune-compromised human patients [2,51–54], snakes might be considered both as spreader or as sentinels for the emergence of zoonotic micro-organisms. Indeed most species of yeasts isolated from snakes have already been established as causative agents of life-threatening infections characterized by high mortality in immune-compromised human patients [2,51–54]. In particular, the World Health Organization classified *C. parapsilosis*, *C. tropicalis* and *C. neoformans* as critical priority pathogens [9]. Whereas, the others (i.e., *Clavispora lusitaniae*, *D. catelunata*, *E. dermatitidis*, *M. caribbica*, *R. mucilaginosa*, *T. asahii*, *W. pararugosa*, *D. hansenii*, *M. silvanorum* and *M. guilliermondii*) are etiological agents of emerging and re-emerging mycoses [2,55–61]. In this study, every yeast species demonstrated distinct virulence traits, been that some were more pathogenic than others, such as *C. gattii/neoformans* species complex, which remain, together with *C. albicans*, one of the main pathogen responsible for invasive yeast infections worldwide [62,63]. Meanwhile, other species herein isolated (i.e., *C. glabrata*, *C. parapsilosis*, *C. tropicalis*) are considered to be emergent, also because of their capacity in acquiring virulent traits according to habitats or hosts in which thrive [62–64].

The production of lipase and hemolysin by all yeasts isolated suggested that these enzymes are part of the metabolic pathway for colonizing the cloaca of healthy snakes, being their production related to the survival of the yeasts in host tissues [64,65]. Conversely, the presence of phospholipase activity in few yeast species herein isolated might be due to a differential yeast pathogenicity, as these enzymes are usually involved in cell membranes damage or disruption of their functions [62–65]. Indeed, mutants lacking these genes are less virulent in murine or in *Galleria mellonella* model [53,66]. However, apart from *W. pararugosa* which is not a phospholipase producer [67], the absence of phospholipase activity in *C. parapsilopsis, C. lusitaniae, D. catelunata, R. mucilaginosa* and *T. asahii* differs from previous results [28, 44,66,68]. This discrepancy may be due to the low number of yeast strains for each species tested or their inability to produce enzymes in snake cloaca, where these yeasts reside [28,69]. The latter hypothesis is supported by the fact that the amount of produced enzymes or biofilm vary according to the host species and environmental conditions [70]. Hence, the higher amount of enzymes or biofilm production in non-venomous snakes that in venomous ones, might suggest that snake venom affects the virulence profiles of yeasts, as in the case of *R. mucillaginosa* isolated from an aglyph snake (i.e., *Z. longissimus*) with a more virulent profile compared to the same species isolated from a mildly venomous opisthoglyphous species (i.e., *M. monspessulanus*). The same differential virulence pattern was recorded comparing *T. asahii* strains isolated from highly venomous solenoglyphous snakes (i.e., *B. arietans*) and mildly venomous species (i.e., *M. monspessulanus*). However, *M. silvanorum* showed a different virulence profile even though it was isolated from two non-venomous species of Italian snakes. This may be due to the fact that the Western whip snake (*H. viridiflavus*) is potentially classified as an opisthoglyphous mildly venomous snake [71], with human cases of envenomation recorded after prolonged bites [71,72]. Overall, data herein presented may suggests that this snake species has salivary compounds analogous to those of snakes that possess Duvernoy’s gland [73]. The absence of any statistically significant differences in virulence profile of *C. tropicalis* isolated from two highly venomous snakes (i.e., *B. arietans*, *N. haje legionis*), further suggest the role of snake venom in rendering less virulent a pathogenic yeast. Indeed, snake venoms are considered as “mini-drug libraries” in which each compound may have potential pharmacological and therapeutical activity [74,75]. Recent data also showed that cobra snakes’ venoms decrease the viability of different *Candida* spp. (i.e., *C. albicans*, *C. tropicalis*, *C. glabrata*) reducing their ability in biofilm production [76]. However, some snake venoms are able to promote the secretion of extracellular phospholipases that may facilitate *Candida* pathogenicity and limit their usefulness as anti-candidal agents [76]. For this reason, the role of snake venom in reducing yeast viability and/or the virulence profile of yeast species, should be better addressed in future studies.

Although this study presents limitations in relation to the number of analyzed strains/per species, the type of samples is quite unique and very rarely investigated, thus representing a novelty for the scientific community. In addition, the state-of-art knowledge enables the researchers to generate new research ideas, contributing to the academia and public health domains. Indeed, data of this study suggests that yeast species isolated from snakes are able to produce hydrolytic enzymes and biofilms, further confirming the role of snakes in spreading pathogenic and virulent organisms [8]. However, the role of these yeast species in producing other virulence factors like secreted aspartic proteinase (SAP) need to be better explored in future studies being that these enzymes are able to cause both mechanical damages (i.e., deteriorate epithelial and mucosal barrier proteins such as collagen, keratin and mucin) and immunological escape (complement, cytokines and immunoglobulins degradation) during the infection process [34]. In *Candida*, SAP is encoded by ten genes SAP1-10 but they were well characterized only for some *Candida* spp. (e.g., *C. albicans*, *C. tropicalis*, *C. parapsilosis* and *C. dubliniensis*) and very low characterized for other yeasts (e.g., *C. glabrata, C. krusei,* and *C. kefyr* [34]. Future comparative studies in the virulence profile as well as in the gene expression between yeasts from venomous/not venomous snakes and yeasts isolated from human/animal clinical cases and environment will be useful to verify the role of venomous in affecting the pathogenic role of yeasts. Nevertheless, studies on usefulness of snake venom to control the growth, biofilm formation, and enzymes production of specific yeast species are warranted, towards the selection of new compounds which could serve as alternatives to available drugs, in order to control fungal infections. Collaborative and integrative efforts are encouraged to better understand the associated virulence factors of yeasts, the physiological or biochemical differential patterns of production in different snake species, in order to develop control and/or prevention measures to mitigate the spread of these virulent yeasts from these reptiles, substantiating the One Health approach.
